# The response to COVID-19 in prisons must consider the broader mental health impacts for people in prison

**DOI:** 10.1177/0004867420937806

**Published:** 2020-06-22

**Authors:** Ashleigh Stewart, Reece Cossar, Mark Stoové

**Affiliations:** 1 Behaviours and Health Risks, Burnet Institute, Melbourne, VIC, Australia; 2 School of Public Health and Preventive Medicine, Monash University, Melbourne, VIC, Australia; 3 Centre for Forensic Behavioural Science, Swinburne University of Technology, Melbourne, VIC, Australia

To the Editor

Prisons are an integral part of the global public health response to coronavirus disease 2019 (COVID-19). In light of typically over-crowded physical environments, prisons operating beyond their capacity and restrictions on freedom of movement, the introduction of COVID-19 in prisons and other custodial settings could be devastating. Effective COVID-19 infection control strategies in custodial settings have seen an emergent emphasis on physical distancing and quarantining (World Health Organization [WHO], 2020). These strategies are crucial to slow COVID-19 transmission; however, they also pose significant risk for people with mental illness in these settings. Rates of severe mental illness and mental health morbidity and mortality are substantially higher among people in prison compared to general populations. Therefore, the response to COVID-19 requires consideration of associated mental health implications to minimise adverse consequences for people in prison.

Strategies to isolate cases from other people in prison may result in additional stressors depending on how people are isolated. Isolation practices may bear similarities, or be perceived as similar, to solitary confinement, with psychological consequences especially damaging for people with preexisting mental illness. The absence of meaningful social contact, environmental stimuli and engagement in purposeful activities through solitary confinement increases the likelihood of severe psychological distress and adverse outcomes post-release (Wildeman and Andersen, 2020). Therefore, opportunities for contactless social engagement and periods spent outdoors need to be integrated into COVID-19 quarantining practices in custodial settings. Similarly, restricting visitations in response to COVID-19 (which resulted in riots and associated fatalities in Italian prisons) will negatively impact psychological well-being, preservation of hopefulness and social connectedness during incarceration (Cochran and Mears, 2013). Adapted communication is needed, such as free and more frequent phone calls and communication via digital platforms with family and friends to alleviate mental health implications.

Prisons and other custodial settings are subject to myriad risk factors for COVID-19 transmission, responses need to avoid unnecessarily harsh restrictions to mitigate COVID-19 transmission. In protecting communities from COVID-19, state and federal policy makers must protect the human rights of highly vulnerable people in custodial settings. There is considerable public dialogue, and associated public health awareness campaigns, about balancing strategies to protect mental health and well-being in the community alongside COVID-19 control imperatives. The same considerations must be applied to measures implemented in prisons to address potential acute and chronic mental health implications and minimise adverse consequences for people in prison who already experience higher rates of serve mental illness.
